# *N*-Cyclo­hexyl-2-oxo-2-phenyl­acetamide

**DOI:** 10.1107/S1600536812023549

**Published:** 2012-05-31

**Authors:** Ze-Jun Jia, Jin-Long Wu

**Affiliations:** a Laboratory of Asymmetric Catalysis and Synthesis, Department of Chemistry, Zhejiang University, Hangzhou, Zhejiang 310027, People’s Republic of China

## Abstract

In the title compound, C_14_H_17_NO_2_, the two carbonyl groups are oriented with respect to each other with a torsion angle of −129.9 (3)°. The cyclo­hexane ring adopts a chair conformation. In the crystal, mol­ecules are linked by N—H⋯O hydrogen bonds into a chain running along the *a* axis.

## Related literature

For related *N*-substituted 2-oxo-2-phenyl­acetamides, see: Boryczka *et al.* (1998 ▶); Dai & Wu (2011 ▶).
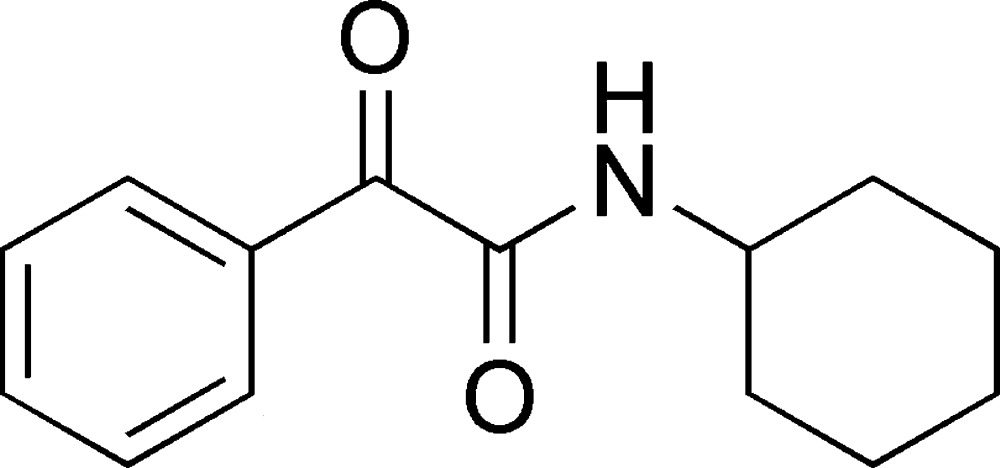


## Experimental

### 

#### Crystal data

C_14_H_17_NO_2_
*M* *_r_* = 231.29
Orthorhombic, 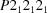

*a* = 9.6942 (4) Å
*b* = 10.4394 (6) Å
*c* = 13.2100 (8) Å
*V* = 1336.87 (12) Å^3^
*Z* = 4
Mo *K*α radiation
μ = 0.08 mm^−1^
*T* = 153 K
0.25 × 0.16 × 0.12 mm

#### Data collection

Bruker SMART 1000 CCD diffractometer
5120 measured reflections
1737 independent reflections
845 reflections with *I* > 2σ(*I*)
*R*_int_ = 0.026

#### Refinement

*R*[*F* ^2^ > 2σ(*F* ^2^)] = 0.036
*wR*(*F* ^2^) = 0.082
*S* = 1.00
1737 reflections
154 parameters
H-atom parameters constrained
Δρ_max_ = 0.08 e Å^−3^
Δρ_min_ = −0.10 e Å^−3^

### 

Data collection: *SMART* (Bruker, 1998 ▶); cell refinement: *SAINT* (Bruker, 1998 ▶); data reduction: *SAINT*; program(s) used to solve structure: *SHELXTL* (Sheldrick, 2008 ▶); program(s) used to refine structure: *SHELXTL* ; molecular graphics: *SHELXTL*; software used to prepare material for publication: *SHELXTL*.

## Supplementary Material

Crystal structure: contains datablock(s) I, global. DOI: 10.1107/S1600536812023549/xu5541sup1.cif

Structure factors: contains datablock(s) I. DOI: 10.1107/S1600536812023549/xu5541Isup2.hkl

Supplementary material file. DOI: 10.1107/S1600536812023549/xu5541Isup3.cml

Additional supplementary materials:  crystallographic information; 3D view; checkCIF report

## Figures and Tables

**Table 1:** Hydrogen-bond geometry (Å, °)

*D*—H⋯*A*	*D*—H	H⋯*A*	*D*⋯*A*	*D*—H⋯*A*
N1—H1⋯O1^i^	0.88	2.02	2.863 (2)	159

Symmetry code: (i) 
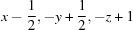
.

**Fig. 1.**
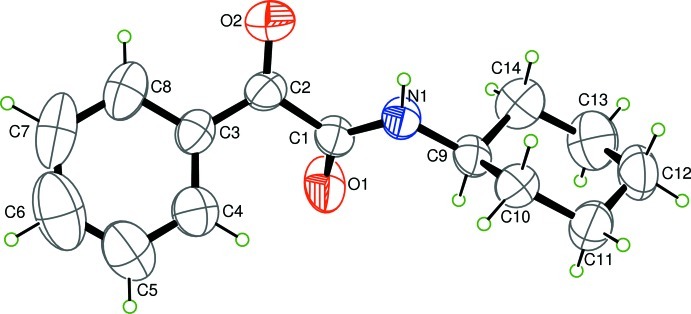
The molecular structure of the title compound with the atom numbering scheme. Displacement ellipsoids are drawn at 40% probability level. H atoms are presented as small spheres of arbitrary radius.

**Fig. 2.**
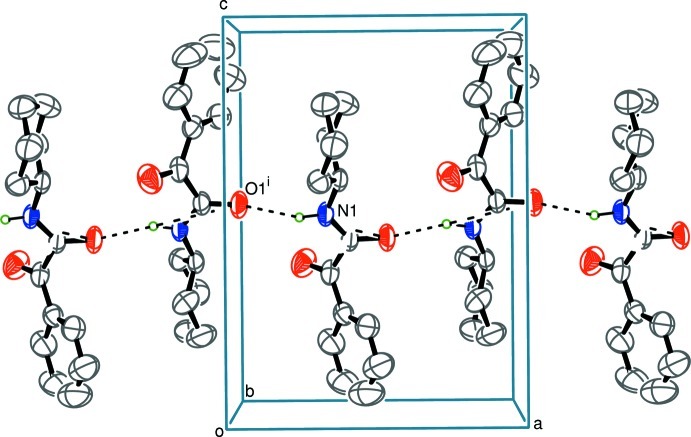
One-dimensional chain molecules of the title compound linked by two adjacent moleculars N—H···O hydrogen bonds (dotted lines). Symmetry code: (i) -0.5 + x, 0.5 - y, 1 - z.

## supplementary crystallographic information

### Comment

Several crystals of N-substituted phenylglyoxamide have been reported, and their great differences of the cystal form due to the N—H···O hydrogen bond linking form have been observed (Boryczka *et al.*, 1998; Dai & Wu, 2011). In our research for exploring the effect rule of the N-substituted group of phenylglyoxamide to the crystal form, we have synthesized the title compound by condensation of phenylglyoxic acid with cyclohexylamine. The structure of the title compound has been characterized by spectroscopic methods and further confirmation by X-ray analysis. We report here its crystal structure. The two carbonyl groups of the molecule are oriented to each other with a torsion angle of -129.9 (3)°. Molecules are linked by intermolecular hydrogen bonds N—H···O into a one-dimensional chain (Fig. 2).

### Experimental

Into a suspension of phenylglyoxylic acid (400 mg, 2.67 mmol) and cyclohexylamine (0.272 ml, 2.38 mmol) in methylene chloride (10 ml), *N*,*N*'-dicyclohexylcarbodiimide (DCC) (540 mg, 2.62 mmol) and 4-(dimethylamino)pyridine (DMAP) (66 mg, 0.54 mmol) was added respectively at room temperature and continuted stirring for 10 h. The reaction mixture was filtered and the filtrate was concentrated under reduced pressure, the residue was purified by colum chromatography (silica gel, 30% of ethyl acetate in hexane) to afford the title compound in 69% yield (379 mg) as a white solid, m.p. 384–385 K. Single crystals suitable for X-ray diffraction of the title compound were grown in a mixture of ethyl acetate and hexane.

### Refinement

The H atoms were placed in calculated positions with C—H = 0.93–0.98 Å, N—H = 0.88 Å and included in the refinement as riding their carrier atoms with *U*_iso_(H) = 1.2*U*_eq_(C,N).

### Crystal data

**Table:**

C_14_H_17_NO_2_	*F*(000) = 496
*M**_r_* = 231.29	*D*_x_ = 1.149 Mg m^−^^3^
Orthorhombic, *P*2_1_2_1_2_1_	Mo *K*α radiation, λ = 0.71073 Å
Hall symbol: P 2ac 2ab	Cell parameters from 6354 reflections
*a* = 9.6942 (4) Å	θ = 3.2–27.5°
*b* = 10.4394 (6) Å	µ = 0.08 mm^−^^1^
*c* = 13.2100 (8) Å	*T* = 153 K
*V* = 1336.87 (12) Å^3^	Block, colourless
*Z* = 4	0.25 × 0.16 × 0.12 mm

### Data collection

**Table:**

Bruker SMART 1000 CCD diffractometer	845 reflections with *I* > 2σ(*I*)
Radiation source: fine-focus sealed tube	*R*_int_ = 0.026
Graphite monochromator	θ_max_ = 27.5°, θ_min_ = 3.3°
φ and ω scans	*h* = −10→12
5120 measured reflections	*k* = −9→13
1737 independent reflections	*l* = −10→16

### Refinement

**Table:**

Refinement on *F*^2^	Primary atom site location: structure-invariant direct methods
Least-squares matrix: full	Secondary atom site location: difference Fourier map
*R*[*F*^2^ > 2σ(*F*^2^)] = 0.036	Hydrogen site location: inferred from neighbouring sites
*wR*(*F*^2^) = 0.082	H-atom parameters constrained
*S* = 1.00	*w* = 1/[σ^2^(*F*_o_^2^) + (0.032*P*)^2^] where *P* = (*F*_o_^2^ + 2*F*_c_^2^)/3
1737 reflections	(Δ/σ)_max_ < 0.001
154 parameters	Δρ_max_ = 0.08 e Å^−^^3^
0 restraints	Δρ_min_ = −0.10 e Å^−^^3^

### Special details

**Table:**

Geometry. All esds (except the esd in the dihedral angle between two l.s. planes) are estimated using the full covariance matrix. The cell esds are taken into account individually in the estimation of esds in distances, angles and torsion angles; correlations between esds in cell parameters are only used when they are defined by crystal symmetry. An approximate (isotropic) treatment of cell esds is used for estimating esds involving l.s. planes.
Refinement. Refinement of F^2^ against ALL reflections. The weighted R-factor wR and goodness of fit S are based on F^2^, conventional R-factors R are based on F, with F set to zero for negative F^2^. The threshold expression of F^2^ > 2sigma(F^2^) is used only for calculating R-factors(gt) etc. and is not relevant to the choice of reflections for refinement. R-factors based on F^2^ are statistically about twice as large as those based on F, and R-factors based on ALL data will be even larger.

### Fractional atomic coordinates and isotropic or equivalent isotropic displacement parameters (Å^2^)

**Table:**

	*x*	*y*	*z*	*U*_iso_*/*U*_eq_	
C1	0.4098 (2)	0.2794 (2)	0.45295 (19)	0.0663 (6)	
C2	0.3397 (2)	0.3534 (2)	0.3683 (2)	0.0684 (7)	
C3	0.3856 (2)	0.3354 (2)	0.2645 (2)	0.0667 (6)	
C4	0.4675 (3)	0.2327 (3)	0.2370 (3)	0.0863 (7)	
H4	0.4986	0.1749	0.2876	0.104*	
C5	0.5046 (4)	0.2127 (4)	0.1389 (4)	0.1220 (11)	
H5	0.5598	0.1408	0.1216	0.146*	
C6	0.4629 (6)	0.2948 (6)	0.0663 (3)	0.1472 (17)	
H6	0.4902	0.2810	−0.0019	0.177*	
C7	0.3812 (4)	0.3986 (5)	0.0898 (4)	0.1328 (15)	
H7	0.3525	0.4561	0.0382	0.159*	
C8	0.3411 (3)	0.4187 (3)	0.1896 (3)	0.0992 (9)	
H8	0.2834	0.4891	0.2064	0.119*	
C9	0.37782 (19)	0.1451 (2)	0.60266 (19)	0.0663 (6)	
H9	0.4801	0.1356	0.5966	0.080*	
C10	0.3146 (2)	0.0142 (2)	0.6000 (2)	0.0774 (7)	
H10A	0.3387	−0.0284	0.5355	0.093*	
H10B	0.2130	0.0217	0.6035	0.093*	
C11	0.3656 (3)	−0.0661 (3)	0.6877 (2)	0.0963 (9)	
H11A	0.3203	−0.1511	0.6857	0.116*	
H11B	0.4663	−0.0794	0.6811	0.116*	
C12	0.3357 (3)	−0.0036 (3)	0.7859 (2)	0.1077 (10)	
H12A	0.3754	−0.0557	0.8413	0.129*	
H12B	0.2346	0.0002	0.7960	0.129*	
C13	0.3938 (3)	0.1297 (3)	0.7912 (2)	0.1173 (11)	
H13A	0.4958	0.1252	0.7919	0.141*	
H13B	0.3635	0.1709	0.8549	0.141*	
C14	0.3468 (3)	0.2104 (3)	0.7015 (2)	0.0926 (8)	
H14A	0.2463	0.2259	0.7067	0.111*	
H14B	0.3941	0.2944	0.7035	0.111*	
N1	0.32939 (16)	0.22256 (17)	0.51791 (15)	0.0709 (6)	
H1	0.2397	0.2315	0.5102	0.085*	
O1	0.53605 (14)	0.27896 (19)	0.45723 (13)	0.1065 (7)	
O2	0.24786 (18)	0.42743 (18)	0.39078 (16)	0.1070 (7)	

### Atomic displacement parameters (Å^2^)

**Table:**

	*U*^11^	*U*^22^	*U*^33^	*U*^12^	*U*^13^	*U*^23^
C1	0.0406 (12)	0.0839 (14)	0.0744 (18)	−0.0046 (11)	0.0022 (12)	0.0129 (15)
C2	0.0468 (12)	0.0696 (14)	0.089 (2)	0.0002 (12)	0.0027 (14)	0.0158 (15)
C3	0.0585 (13)	0.0672 (14)	0.074 (2)	−0.0106 (13)	−0.0036 (14)	0.0135 (15)
C4	0.0808 (16)	0.0898 (18)	0.088 (2)	−0.0109 (17)	0.0028 (17)	0.0012 (17)
C5	0.137 (3)	0.125 (3)	0.104 (3)	−0.020 (2)	0.027 (3)	−0.016 (3)
C6	0.188 (4)	0.167 (4)	0.087 (3)	−0.077 (4)	0.014 (3)	−0.015 (3)
C7	0.158 (3)	0.148 (3)	0.093 (3)	−0.044 (3)	−0.029 (3)	0.054 (3)
C8	0.107 (2)	0.0941 (18)	0.097 (3)	−0.0214 (17)	−0.015 (2)	0.030 (2)
C9	0.0369 (10)	0.0915 (15)	0.0706 (18)	−0.0062 (11)	0.0024 (12)	0.0167 (16)
C10	0.0801 (15)	0.0763 (16)	0.0757 (19)	0.0060 (14)	−0.0005 (16)	0.0040 (15)
C11	0.0945 (19)	0.0939 (17)	0.101 (3)	0.0056 (16)	0.0059 (19)	0.0259 (19)
C12	0.102 (2)	0.144 (3)	0.077 (3)	0.005 (2)	0.005 (2)	0.037 (2)
C13	0.131 (2)	0.145 (3)	0.076 (2)	−0.012 (2)	−0.026 (2)	−0.007 (2)
C14	0.0999 (19)	0.0907 (17)	0.087 (2)	−0.0083 (17)	−0.0252 (18)	−0.0073 (18)
N1	0.0344 (8)	0.0975 (13)	0.0809 (15)	−0.0005 (9)	0.0012 (9)	0.0273 (12)
O1	0.0367 (8)	0.1821 (17)	0.1007 (15)	−0.0129 (10)	−0.0007 (9)	0.0534 (14)
O2	0.0890 (12)	0.1142 (14)	0.1178 (17)	0.0351 (12)	0.0181 (13)	0.0217 (14)

### Geometric parameters (Å, º)

**Table:**

C1—O1	1.226 (2)	C9—C14	1.504 (3)
C1—N1	1.302 (2)	C9—H9	1.0000
C1—C2	1.519 (3)	C10—C11	1.512 (3)
C2—O2	1.216 (3)	C10—H10A	0.9900
C2—C3	1.454 (3)	C10—H10B	0.9900
C3—C4	1.383 (3)	C11—C12	1.481 (4)
C3—C8	1.386 (3)	C11—H11A	0.9900
C4—C5	1.360 (4)	C11—H11B	0.9900
C4—H4	0.9500	C12—C13	1.502 (4)
C5—C6	1.348 (5)	C12—H12A	0.9900
C5—H5	0.9500	C12—H12B	0.9900
C6—C7	1.378 (5)	C13—C14	1.524 (3)
C6—H6	0.9500	C13—H13A	0.9900
C7—C8	1.390 (5)	C13—H13B	0.9900
C7—H7	0.9500	C14—H14A	0.9900
C8—H8	0.9500	C14—H14B	0.9900
C9—N1	1.459 (3)	N1—H1	0.8800
C9—C10	1.498 (3)		
			
O1—C1—N1	124.4 (2)	C11—C10—H10A	109.5
O1—C1—C2	118.8 (2)	C9—C10—H10B	109.5
N1—C1—C2	116.70 (18)	C11—C10—H10B	109.5
O2—C2—C3	122.5 (2)	H10A—C10—H10B	108.1
O2—C2—C1	118.1 (3)	C12—C11—C10	111.3 (2)
C3—C2—C1	119.5 (2)	C12—C11—H11A	109.4
C4—C3—C8	118.5 (3)	C10—C11—H11A	109.4
C4—C3—C2	121.6 (2)	C12—C11—H11B	109.4
C8—C3—C2	119.8 (3)	C10—C11—H11B	109.4
C5—C4—C3	121.4 (3)	H11A—C11—H11B	108.0
C5—C4—H4	119.3	C11—C12—C13	112.1 (3)
C3—C4—H4	119.3	C11—C12—H12A	109.2
C6—C5—C4	120.1 (4)	C13—C12—H12A	109.2
C6—C5—H5	120.0	C11—C12—H12B	109.2
C4—C5—H5	120.0	C13—C12—H12B	109.2
C5—C6—C7	120.8 (4)	H12A—C12—H12B	107.9
C5—C6—H6	119.6	C12—C13—C14	111.3 (3)
C7—C6—H6	119.6	C12—C13—H13A	109.4
C6—C7—C8	119.5 (4)	C14—C13—H13A	109.4
C6—C7—H7	120.2	C12—C13—H13B	109.4
C8—C7—H7	120.2	C14—C13—H13B	109.4
C3—C8—C7	119.7 (3)	H13A—C13—H13B	108.0
C3—C8—H8	120.2	C9—C14—C13	111.4 (2)
C7—C8—H8	120.2	C9—C14—H14A	109.4
N1—C9—C10	110.88 (19)	C13—C14—H14A	109.4
N1—C9—C14	110.53 (19)	C9—C14—H14B	109.4
C10—C9—C14	110.6 (2)	C13—C14—H14B	109.4
N1—C9—H9	108.2	H14A—C14—H14B	108.0
C10—C9—H9	108.2	C1—N1—C9	124.46 (16)
C14—C9—H9	108.2	C1—N1—H1	117.8
C9—C10—C11	110.8 (2)	C9—N1—H1	117.8
C9—C10—H10A	109.5		
			
O1—C1—C2—O2	−129.9 (3)	C2—C3—C8—C7	−177.8 (3)
N1—C1—C2—O2	48.6 (3)	C6—C7—C8—C3	1.1 (5)
O1—C1—C2—C3	49.1 (3)	N1—C9—C10—C11	179.74 (19)
N1—C1—C2—C3	−132.4 (2)	C14—C9—C10—C11	−57.3 (3)
O2—C2—C3—C4	−165.7 (2)	C9—C10—C11—C12	57.3 (3)
C1—C2—C3—C4	15.4 (3)	C10—C11—C12—C13	−55.3 (3)
O2—C2—C3—C8	10.9 (3)	C11—C12—C13—C14	53.3 (4)
C1—C2—C3—C8	−168.0 (2)	N1—C9—C14—C13	178.8 (2)
C8—C3—C4—C5	0.0 (4)	C10—C9—C14—C13	55.6 (3)
C2—C3—C4—C5	176.7 (2)	C12—C13—C14—C9	−53.3 (3)
C3—C4—C5—C6	0.9 (5)	O1—C1—N1—C9	−3.0 (4)
C4—C5—C6—C7	−0.9 (6)	C2—C1—N1—C9	178.6 (2)
C5—C6—C7—C8	−0.1 (6)	C10—C9—N1—C1	−125.1 (2)
C4—C3—C8—C7	−1.1 (4)	C14—C9—N1—C1	111.8 (2)

### Hydrogen-bond geometry (Å, º)

**Table:**

*D*—H···*A*	*D*—H	H···*A*	*D*···*A*	*D*—H···*A*
N1—H1···O1^i^	0.88	2.02	2.863 (2)	159

Symmetry code: (i) *x*−1/2, −*y*+1/2, −*z*+1.
